# Should we abandon hormonal therapy in endometrial cancer? Outcomes of recurrent and metastatic endometrial cancer treated with systemic progestins

**DOI:** 10.1002/cam4.6276

**Published:** 2023-07-07

**Authors:** A. Kulkarni, N. M. Andrews Wright, A. N. Forget, T. Ramsay, R. Mallick, J. I. Weberpals

**Affiliations:** ^1^ Department of Obstetrics and Gynecology, Division of Gynecologic Oncology, Faculty of Medicine University of Ottawa Ottawa Ontario Canada; ^2^ Ottawa Hospital Research Institute Ottawa Ontario Canada; ^3^ School of Epidemiology, Public Health and Preventative Medicine University of Ottawa Ottawa Ontario Canada

**Keywords:** death, metastasis, recurrence, skin neoplasms

## Abstract

**Purpose:**

The objective of this study is to determine primary survival endpoints in women with recurrent and metastatic endometrial carcinoma (RMEC) treated with progestins.

**Methods:**

A retrospective chart review was conducted at The Ottawa Hospital using electronic medical records. Inclusion criteria were a diagnosis of RMEC between 2000 and 2019, endometrioid histology, and ≥one line of progestin treatment. Progression‐free survival (PFS) and overall survival (OS) were estimated using the Kaplan–Meier method.

**Results:**

Of 2342 cases reviewed, 74 met inclusion criteria. Sixty‐six (88.0%) patients received megestrol acetate and 9 (12.0%) received a progestin alternative. The distribution of tumors by grade was: 1: 25 (33.3%), 2: 30 (40.0%), and 3: 20 (26.7%). The PFS and OS for the entire study sample was 14.3 months (95% CI 6.2–17.9) and 23.3 months (14.8–36.8), respectively. The PFS for patients with Grade 1–2 RMEC was 15.7 months (8.0, 19.5), compared to 5.0 months (3.0, 23.0) with Grade 3 disease. The OS for patients with Grade 1–2 versus Grade 3, was 25.9 months (15.3, 40.3) versus 12.5 months (5.7, 35.9), respectively. Thirty‐four (45.9%) and 40 (54.1%) patients were treated with 0 and ≥1 line of chemotherapy. The PFS for chemotherapy‐naïve patients was 17.9 months (14.3, 27.0), versus 6.2 months (3.9, 14.8) following ≥1 line of treatment. The OS was 29.1 months (17.9, 61.1) for chemotherapy‐naïve patients versus 23.0 months (10.5, 37.6) for patients previously exposed.

**Conclusions:**

This real‐world data on RMEC suggests there is a role for progestins in select subgroups of women. The PFS for chemotherapy‐naïve patients was 17.9 months (14.3, 27.0), versus 6.2 months (3.9, 14.8) following ≥1 line of treatment. The OS was 29.1 months (17.9, 61.1) for chemotherapy‐OS was 29.1 months (17.9, 61.1) for chemotherapy‐naïve patients versus 23.0 months (10.5, 37.6) for patients previously exposed.

## Abstract 2

This real‐world data on recurrent and metastatic endometrial carcinoma (RMEC) suggests there is a role for progestins in select subgroups of women. The PFS for chemotherapy‐naïve patients was 17.9 months (14.3, 27.0), versus 6.2 months (3.9, 14.8) following>=1 line of treatment. The OS was 29.1 months (17.9, 61.1) for chemotherapy‐OS was 29.1 months (17.9, 61.1) for chemotherapy‐naïve patients versus 23.0 months (10.5, 37.6) for patients previously exposed.

## 1 INTRODUCTION

Endometrial carcinoma (EC) is the fourth most common cancer in women and is increasing in incidence in Western countries.^1^ The most common subtype represents approximately 80% of cases, characterized by Grade 1–2 endometrioid histology and the expression of estrogen (ER) and/or progesterone receptors (PR).^2^ While treatment for early‐stage disease is highly effective, 7%–15% of patients with Stage I‐II disease and 40% of patients with advanced‐stage disease will recur.^3^, ^4^ Options for systemic therapy for recurrent and metastatic EC (RMEC) include chemotherapy, immunotherapy and hormonal therapy^5^ with a wide range of response rates reported.^6^ Recent treatment strategies have focused on immunotherapy and novel targeted therapies with key Phase II‐III trials summarized in Table S1 and Table S2.

Hormonal therapies are frequently recommended for patients with Grade 1–2 endometrioid adenocarcinoma with low tumor burden.^5^ Without any head‐to‐head trials, treatment with hormonal therapy in RMEC remains at the discretion of the treating oncologist.^7^ Hormonal therapy with progestins have been commonly used for patients with RMEC due to a well‐tolerated toxicity profile as women with RMEC are often advanced in age with multiple comorbidities.^8^ Since the 1960s, progestins have represented a viable therapeutic option, with response rates from 15 to 33%, and median progression‐free survival (PFS) ranging from 2.5 to 4 months.^9^

However, previous studies have used different progression and evaluation criteria than would be acceptable by today's standards, making modern‐day comparisons impossible.^10^, ^11^ Today, PFS and overall survival (OS) are the gold standard efficacy outcomes, and the value of predictive tumor characteristics and biomarkers are now better understood. To place progestin use in RMEC in a modern context, a real‐world evaluation with current evaluation criteria is required. In this single‐center retrospective cohort study on women with RMEC, we evaluated primary clinical outcomes of progestin therapy.

## 2 METHODS

### 2.1 Study design

This is a retrospective, single‐center cohort study including patients treated for RMEC at The Ottawa Hospital Cancer Centre (TOHCC), Ottawa, Canada. Patients were followed until the data cutoff of January 2022. The study was approved by the Ottawa Health Science Network Research Ethics Board (#20190797‐01H). Data were reported according to STROBE reporting guidelines for cohort studies.^12^

All patients with a primary diagnosis of EC between January 1, 2000 and May 31, 2019 at TOHCC were reviewed. Inclusion criteria were adults (≥ 18 years of age) with a diagnosis of EC, endometrioid histology by initial pathology report (or mixed histology with predominantly endometrioid component >50%), documentation of recurrent or metastatic disease not amenable to surgical resection or radiation therapy, and at least one line of progestin‐based treatment in the recurrent or metastatic setting. Exclusion criteria were primary nonmetastatic disease, a diagnosis of a concurrent malignancy within 5 years and patients whose primary endpoint data was unavailable due to loss to follow‐up.

### 2.2 Variables and data sources

Patient identification was conducted using an automated database; the electronic medical records of all patients between January 1, 2000 and May 31, 2019 were reviewed for a diagnosis code of EC (ICD‐10 C54.1). Further selection of patients with endometrioid and mixed histological subtypes (ICD‐O 3 codes 83803, 83813, 83823) was undertaken followed by manual review of inclusion/exclusion criteria by three study investigators (AK, AF, and NW). Based on the number of treated RMEC cases per year at TOHCC, a sample of up to 120 patients was permitted.

Data were sourced from the electronic medical records. Collected data points are found in Table S3 and include demographic information, molecular and pathological characteristics, comorbidities, progestin type and dose, and prior treatment history. The primary outcome was PFS, defined as the time from initiation of progestin‐based therapy until progression of disease by documented clinical progression, imaging, biopsy, or death from any cause. OS was a secondary outcome, defined as the time from initiation of progestin‐based therapy until death from any cause.

Response rates were defined as: complete response, partial response, stable disease, or progressive disease, based on radiologic or clinical assessment documented in the patient chart. The overall response rate (ORR) was defined as the sum of those who had a complete and partial response.

### 2.3 Statistical analysis

Descriptive statistics were completed on demographic and disease characteristics data. Median time to PFS and OS were estimated using the Kaplan–Meier method and were stratified by grade and prior treatment. All statistical analysis was performed using SAS 9.4, SAS Institute Inc, Cary, NC, USA. Plots were created using RStudio, version 1.2.1335.

## 3 RESULTS

### 3.1 Patient clinical characteristics

**FIGURE 1:**
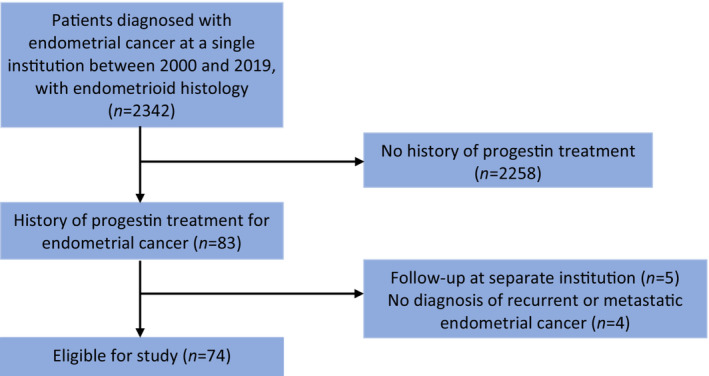
Patient Flow Diagram.

Of 2342 cases reviewed with a primary diagnosis of EC between January 2000 to May 2019, 74 met the inclusion criteria (Figure 1). Automated database review identified 2342 cases of endometrial cancer with endometrioid histology at TOHCC between 2000 and 2019. Of these, 2258 had no history of progestin treatment. From the 83 patients with prior progestin treatment for endometrial cancer, five did not have adequate follow‐up data and four did not have recurrent or metastatic disease. The remaining 74 patients were included in the study. The clinicopathological characteristics of the study sample are outlined in Table 1. The mean age of women at the time of diagnosis of endometrial cancer was 66.8 years and the range of follow‐up was 0–199 months. One participant had a long‐term response of 199 months at the time of data evaluation and only three patients had follow‐up exceeding 100 months.

**TABLE 1:** Clinicopathological Characteristics.

Total number of patients	74
Age at diagnosis [years] (mean [SD])	66.8 (13.4)
Histology (number [%])	
Endometrioid	72 (97.3)
Mixed endometrioid/clear cell	1 (1.4)
Mixed endometrioid/serous/mucinous	1 (1.4)
Histology grade (number [%])	
1	25 (33.8)
2	29 (39.2)
3	20 (27.0)
Stage at diagnosis (number [%])	
1	26 (35.1)
2	13 (17.6)
3	8 (10.8)
4	18 (24.3)
Unstaged (no evidence of metastatic disease on imaging)	9 (12.2)
Number of prior lines of systemic treatment (number [%])	
0	34 (45.9)
1	34 (45.9)
2	5 (6.8)
3	1 (1.4)
Time from initial diagnosis to starting progesterone treatment [months] (mean [SD])	32.4 (31.3)
Progesterone treatment used for de novo metastatic versus recurrent disease (number [%])	
De novo metastatic	7 (9.5)
Recurrent	67 (90.5)

The distribution by tumor grade was: 1: 25 patients (33.8%); 2: 29 (39.2%); and 3: 20 (27.0%). Nearly half the cohort (34, 45.9%) were chemotherapy‐naïve prior to beginning progestin treatment and 40 (54.1%) received ≥1 prior line of chemotherapy. Most patients (67 [90.5%]) were on progestins for the treatment of recurrent EC, in contrast to 7 (9.5%) treated for de novo metastatic disease. The median time from initial diagnosis of EC to starting a progestin treatment was 26 months (range 0–145).

### 3.2 Progestin treatment and tolerability

In this study, 65 (87.8%) women were treated with megestrol acetate, 7 (9.5%) with medroxyprogesterone, and 2 (2.7%) with an unspecified progestin. The characteristics of progestin treatment are outlined in Table 2. Of women treated with megestrol acetate, the majority (64.6%) were started on an initial dose of 80 mg twice daily (or 160 mg once daily). Nearly half (48.6%) of patients did not require dose modifications during the follow‐up period; 5 (6.8%) required a dose reduction due to side effects and 2 (2.7%) had an increase. Data on dose modifications were not available for 31 (41.9%) patients. Of all patients, 41 (55.4%) stopped taking progestin treatment due to disease progression and 20 (27.0%) died while on treatment due to advanced disease. Only two women stopped progestin due to adverse events; 1 (1.4%) had intolerable side effects, and another 1 (1.4%) experienced an acute pulmonary embolism. Finally, 11 (14.9%) women were still receiving progestin treatment at the time of data abstraction.

**TABLE 2:** Characteristics of Progestin Treatment.

Total number of patients	74
Total number tested for hormone receptor status (number [%])	
Hormone receptor status positive (number [%])	50 (67.6)
ER	48 (96.0)
PR	44 (88.0)
Both ER/PR	41 (82.0)
Type of progestin used (number [%])	
Megestrol acetate	65 (87.8)
Medroxyprogesterone	7 (9.5)
Other	2 (2.7)
Initial Dose of Megestrol Acetate (number [%])	
40 mg po bid	5 (6.8)
80 mg po bid	31 (41.9)
160 mg po od	11 (14.9)
160 mg po bid	8 (10.8)
320 mg po od	1 (1.4)
Unknown	9 (12.2)
Change in Dose of Progestin (number [%])	
Decrease	5 (6.8)
Increase	2 (2.7)
None	36 (48.6)
Unknown	31 (41.9)
Reason for stopping progestin (number [%])	
Death	20 (27.0)
Progression of disease	41 (55.4)
Side effects	2 (2.7)
Currently taking	11 (14.9)

Fifty of 74 tumors (67.6%) (either from surgery or tissue biopsy) were tested for hormone receptor status; 56% of the Grade 1 tumors, 83% of the Grade 2 tumors and 60% of the Grade 3 tumors. All the tested specimens were positive for either ER or PR. Of the 50 tumors, 48 (96.0%) were ER+, 44 (88.0%) were PR+, and 41 (82.0%) were positive for both.

Table 3 demonstrates an unplanned evaluation of treatment response. 5 (6.8%) patients had a complete response to progestin treatment, 8 (10.8%) had a partial response, and 23 (31.1%) had stable disease. This resulted in an ORR of 17.6%. All 5 complete responders demonstrated Grade 1 or 2 histology.

**TABLE 3:** Response Rates to Progestin.

Best Response	*n* (%)
Overall Response Rate (ORR)	13 (17.6)
Complete response	5 (6.8)
Partial response	8 (10.8)
Stable disease	22 (29.7)
Progressive disease	27 (36.5)
Not assessed	12 (16.2)

### 3.3 Survival outcomes

The median PFS for the study sample was 14.3 months (95% CI 6.2–17.9) and the median OS was 23.3 months (95% CI 14.8–36.8) (Figure S1). The duration of treatment with systemic progestins varied greatly. One patient experienced early tumor progression within a month of starting treatment while another remains on systemic progestins after 14 years of treatment, with no evidence of disease progression.

**FIGURE 2:**
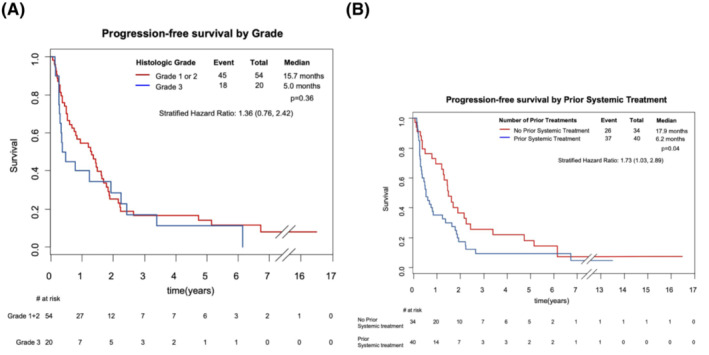
(A) Kaplan–Meier curves demonstrating PFS comparing: (A) grade 1 and 2 histology to Grade 3, and (B) patients treated with 1 or more previous lines of chemotherapy to those who were chemotherapy‐naïve. Estimates provided are based on Cox proportional hazards regression model stratifying for grade and number of prior lines of systemic treatment.

A subset analysis was done according to histologic grade (Figure 2); the median PFS for patients with Grade 1 or 2 disease was 15.7 months (8.0, 19.6) compared to 5.0 months (3.0, 23.0) for Grade 3 disease.

The proportion of patients was evenly divided between those who were chemotherapy‐naïve before receiving progestins and those receiving progestins following ≥1 line of chemotherapy for EC. The distribution of clinical characteristics of these two groups is shown in Table 4. There was a significant difference in the mean age of diagnosis for patients who were chemotherapy‐naïve compared to those who were previously exposed at 74.1 years (SD = 12.6) versus 60.6 years (SD = 10.7), respectively, (*p* < 0.0001). The groups did not otherwise significantly differ according to histology, grade, or stage at diagnosis.

**TABLE 4:** Patient characteristics comparing those who are chemotherapy‐naïve to those who have received at least one previous line of chemotherapy.

Variable	0 previous lines (*n* = 34)	> = 1 previous line (*n* = 40)	*p*‐value
Age at diagnosis (years) (mean [SD])	74.1 (12.6)	60.6 (10.7)	<0.0001
Histology (number [%])			0.42
Endometrioid	34 (100)	38 (95.0)	
Mixed endometrioid/clear cell	0 (0.0)	1 (2.5)	
Mixed endometrioid/serous/mucinous	0 (0.0)	1 (2.5)	
Histology grade (number [%])			0.08
1	16 (47.1)	9 (22.5)	
2	10 (29.4)	19 (47.5)	
3	8 (23.5)	12 (30.0)	
Stage at diagnosis (number [%])			0.07
1	11 (32.4)	15 (37.5)	
2	6 (17.6)	7 (17.5)	
3	2 (5.9)	6 (15.0)	
4	7 (20.6)	11 (27.5)	
Unstaged (no evidence of metastatic disease on imaging)	8 (23.5)	1 (2.5)	
Median PFS (months)	17.9 (14.3, 27.0)	6.2 (3.9, 10.0)	0.04
Median OS (months)	29.1 (17.9, 61.1)	15.0 (10.1, 35.9)	0.45

A subgroup analysis was performed to compare the PFS between chemotherapy‐naïve and chemotherapy‐exposed patients. (Figure 2). The median PFS for chemotherapy‐naïve patients was 17.9 months (14.3, 27.0), compared to 6.2 months (3.9, 10.0) for patients who had received ≥1 prior lines of treatment.

**FIGURE 3:**
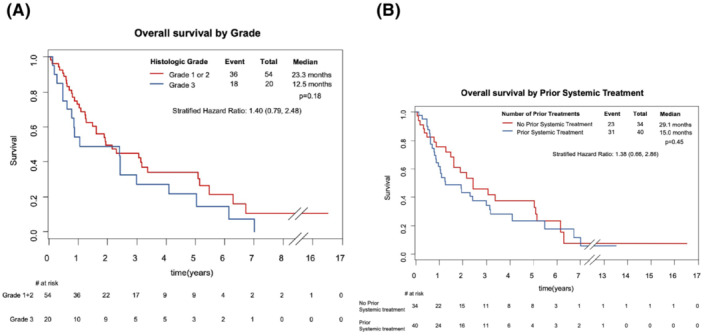
Kaplan–Meier curves demonstrating OS comparing: (A) Grade 1 and 2 histology to Grade 3, and (B) patients treated with 1 or more previous lines of chemotherapy to those who were chemotherapy‐naïve. Estimates provided are based on Cox proportional hazards regression model stratifying for age, grade and number of prior lines of systemic treatment.

Median OS was favorable in women with Grade 1 or 2 disease (25.9 months; 15.0, 40.3) compared to women with Grade 3 disease (12.5 months; 5.7, 35.9). For chemotherapy‐naïve patients, the median OS was 29.1 months (17.9, 61.1) compared to 15.0 months (10.1, 35.9) for those who were previously exposed (Figure 3).

## 4 DISCUSSION

This study on patients with RMEC treated with systemic progestins is the largest reported in over 20 years. In all patients, we report a median PFS of 14.3 months (95% CI 6.2–17.9), median OS of 23.3 months (95% CI 14.8–36.8) and an ORR of 17.6%. In subgroup analysis, for chemotherapy‐naïve patients versus patients previously exposed to ≥1 line of chemotherapy, we found a median PFS of 17.9 months and 6.2 months, respectively. The ORR of 17.6% was in keeping with previously reported ORRs ranging from 18 to 26% in earlier clinical trials.^13^

The PFS found our study was higher compared to prior reports.^13^ Two small, interventional Phase II studies used progestins in their control arms in the first‐ and second‐line setting of RMEC.^14^, ^15^ The most comparable single‐study population to our cohort is a small, interventional Phase II trial with oral megestrol acetate as the control arm in ER+ RMEC patients (*n* = 35) not eligible for treatment with surgery or radiotherapy alone^14^; the ORR of megace was 35.3% with a median PFS of 40 weeks (90% CI: 16.3–64.0). Another Phase II trial had 52 patients in the comparator arm treated with progestins alone, showing an ORR of 4% and a PFS of 1.9 months (95% CI: 1.9–2.3).^15^ However, 38% of patients had more aggressive histological subtypes, including serous carcinoma and carcinosarcoma and over half of the patients had Grade 3 disease, likely contributing to the poor outcomes. A recent meta‐analysis conducted a weighted calculation among studies where unselected patients were treated with first‐line single‐agent progestin and arrived at a pooled ORR of 23.3%, a PFS of 2.9 months and OS of 9.2 months.^13^ This study included case reports as well as trial data, where variable response evaluation criteria was used and patient tumor characteristics were inconsistently reported, compromising the ability to make meaningful comparisons.

Other clinical trials examining different doses of progestins in RMEC have reported PFS in the range of 2.5–3.2 months and OS of 7.0–11.1 months.^16^, ^17^ Most prior studies evaluating hormonal treatment in RMEC included several therapeutic agents such as progestins in addition to selective estrogen receptor modulators, aromatase inhibitors, synthetic steroid derivatives and GnRH analogs.^13^ Trials examining various combinations and schedules of progestins with tamoxifen, a presumed enhancer of PR expression, in RMEC have reported median PFS in the range of 2.7–3 months and OS of 13–14 months.^18^, ^19^ Two of these studies described durable responses in the patients who responded to treatment (27%), with a median response duration of 28 months,^19^ and 6.5 to 27 months, respectively.^17^ The total size of these comparable studies was small, ranging from 56 to 145 patients, and included all grades and histological subtypes, though endometrioid histology was the most common. All analyses were conducted in patients with recurrent, metastatic, or less frequently, Stage III advanced disease, and patients who were naïve to systemic therapy (chemotherapy or hormonal therapy) with disease that could not be treated curatively.

The chemotherapy‐naïve population is of significant real‐world interest because this group of patients tends to be older and of lower performance status compared to those who receive chemotherapy. In the chemotherapy‐naïve population, our documented PFS (17.9 months) and OS (29.1 months) is considerably higher than the range documented by prior studies in a similar patient population. This is likely attributed to the homogenous nature of our cohort, which exhibits favorable prognostic features such as endometrioid histology, low tumor grade and hormone receptor positivity. Of the 50/74 patients in our cohort tested for ER/PR status, we found a high rate of hormone receptor positivity. In accordance with previous studies, higher levels of ER and PR expression have been associated with improved PFS and OS.^8^ Consistent with prior reports, we found that progestins are well‐tolerated. Only 2.7% of patients in our cohort stopped treatment due to intolerance or side effects. This favorable toxicity profile makes progestins an attractive treatment option for older patients with comorbidities.^17^, ^20^ The most significant toxicity in our cohort was venous thromboembolism, which occurred in only one patient.

While reports describing other therapeutics are not directly comparable due to study design and population differences, the PFS findings from practice‐changing chemotherapy studies are not dissimilar to the findings in the current study. GOG 209 was a non‐inferiority study comparing carboplatin plus paclitaxel to the then‐standard paclitaxel‐doxorubicin‐cisplatin regimen in chemotherapy‐naïve patients with RMEC. The reported PFS of 13–14 months was relatively comparable to the PFS in our chemotherapy‐naïve (first‐line) subgroup (17.9 months). However, the OS of 37–41 months is numerically greater than our reported OS in the chemotherapy‐naïve group of 29.1 months. It is noteworthy that patients with recurrent EC represented only 27.3% of the sample in GOG 209, whereas our study had 90.5% with recurrent disease which may account for the OS difference between the two studies.^21^

A landmark trial in recurrent endometrial carcinoma, Keynote‐775, studied the use of pembrolizumab plus lenvatinib versus investigator's choice of single‐agent chemotherapy in patients who had received at least one line of platinum‐based chemotherapy. In the overall population, the PFS for women treated with pembrolizumab/lenvatinib was 7.2 months, compared to 3.8 months for patients treated with chemotherapy. While the PFS in the pembrolizumab/lenvatinib arm was not dissimilar to our calculated PFS (6.2 months), the study populations are not necessarily comparable.^22^ The KEYNOTE‐775 study included over 30% serous and clear cell histologies and nearly a quarter of patients had high grade endometrioid histology. However, it is recognized that the combination of pembrolizumab and lenvatinib may be associated with significant toxicity and thus may not be the first choice of treatment for elderly and frail patients with recurrent disease.^22^ In our study, we noted that hormonal agents were often considered due to the favorable side effect profile in patients who were otherwise unable to tolerate other systemic therapies.

Retrospective studies permit real‐world observation but are not without their limitations. Follow‐up medical record data was not complete for all patients, and follow‐up frequency during treatment with systemic progestins was at the treating physician's discretion. Some patients were routinely imaged every 3 months, while others had imaging only if symptomatic. This may lead to less precision in the declaration of a PFS outcome, compared to prospective studies, given that progression on imaging often appears prior to patients experiencing symptoms. Furthermore, we did not have consistent information on the volume of disease patients had prior to starting progestin treatment. Current guidelines suggest consideration of progestin for patients with low tumor volume or indolent growth; however, there are no clear guidelines in the literature for specific criteria that qualify RMEC patients for progestin treatment. This could have led to selection bias due to physicians being more likely to choose patients for progestin treatment if they had lower disease burden, leading to an overestimation of PFS.

Our study highlights the importance of drug development in a common woman's cancer such as endometrial cancer. New clinical trials in RMEC have not kept pace with the increasing incidence and mortality of the disease. In an era of personalized medicine, despite emerging recognition of distinct biological subtypes, until very recently, the management of endometrial cancer has not considered underlying molecular characteristics. Furthermore, endometrial carcinoma is an outlier among other cancer types, particularly among hormonally driven cancers such as breast and prostate cancer, where so few robust therapeutic options are available for advanced disease. As a result, the landscape of clinical trials has had a void in the development of novel hormonal agents in recent years.

The current understanding of endometrial carcinoma has evolved into a molecular classification of tumor subtypes each driven by genetic alterations and activation/inactivation of specific pathways. Given that RMEC can no longer be considered as one disease, one would envision a biomarker approach to systemic therapy guided by a patient's tumor molecular profile. Although the use of immunotherapy has changed the face of systemic therapy for RMEC particularly in patients with mismatch repair deficiency, the adverse event rate and toxicity profile of this combination therapy may preclude its use in a significant proportion of patients.^22^ The future treatment of RMEC would benefit from a customized approach based on the pathologic and molecular characteristics of the disease being exploited to their fullest.^23^, ^24^ With maturing biomarker data, clinical trials that take advantage of new targeted therapies will likely add to the current armamentarium of existing treatment and improve the outcomes of patients with advanced or recurrent disease.

## 5 CONCLUSIONS

This study suggests there is a role for progestins in select subgroups of women with RMEC. Active areas of clinical research in endometrial cancer should comprise both the identification of patients most likely to benefit from hormonal therapy as well as the evaluation of more modern hormonal compounds.

## AUTHOR CONTRIBUTIONS

**Anjali Kulkarni:** Data curation (equal); formal analysis (equal); investigation (equal); visualization (equal); writing – original draft (equal); writing – review and editing (equal). **N. M. Andrews Wright:** Data curation (equal); methodology (equal); project administration (equal); resources (equal); visualization (equal). **Alyssa Forget:** Data curation (equal); investigation (equal); methodology (equal); resources (equal). **Tim Ramsay:** Investigation (equal); methodology (equal); software (equal). **Ranjeeta Mallick:** Formal analysis (equal). **J. I. Weberpals:** Conceptualization (equal); data curation (equal); formal analysis (equal); funding acquisition (equal); investigation (equal); methodology (equal); project administration (equal); supervision (equal); validation (equal); visualization (equal); writing – original draft (equal); writing – review and editing (equal).

## FUNDING INFORMATION

This research was conducted with support from AstraZeneca Canada Inc. J.I. W. has received funding from AstraZeneca for the submitted work; has participated in AstraZeneca advisory boards, outside the submitted work; has received honoraria from AstraZeneca, outside the submitted work. A.K., A.N.F., N.M.A.W., R.M., T.R., declare no conflicts of interest relevant to this publication.

## Supporting information


Data S1:

## Data Availability

Data sharing is not applicable to this article as no new data were created or analyzed in this study.
