# Supplementary material for: Should we abandon hormonal therapy in endometrial cancer? Outcomes of recurrent and metastatic endometrial cancer treated with systemic progestins
Source: Cancer Med. 2023 Jul 7;12(15):16173–80. doi: 10.1002/cam4.6276 (PMC10469659; doi:10.1002/cam4.6276)
Supplement: Supplementary file 1 — Data S1: [file CAM4-12-16173-s001.docx]

**Table S1: Response rates of RMEC to systemic therapies (Phase II-III studies conducted from 2012 to present)**

| **Study** | **Study Population** | **Intervention** | **Overall Response Rate** | **PFS** |
| --- | --- | --- | --- | --- |
| Makker et al, 2022 | Patients with advanced endometrial cancer, previously received platinum-based chemotherapy | Pembrolizumab and lenvatinib | 31.9% | 7.2 months |
| Rubinstein et al, 2020 | Advanced endometrial cancer of any grade, prior chemotherapy, demonstration of PI3K pathway activation | LY3023414 (dual PI3K/mTOR inhibitor) | 16% | Not reported |
| Miller et al, 2020 | Stage III-IV or recurrent endometrial cancer | Doxorubicin and cisplatin, followed by paclitaxel vs. carboplatin and paclitaxel | Not reported | 14 months vs. 13 months |
| Lorusso et al, 2019 | Stage III-IV or recurrent endometrial cancer | Carboplatin/paclitaxel standard dose for 6-8 cycles vs. carboplatin/paclitaxel and bevacizumab | 53.1% vs 74.4% | 10.5 vs 13.7 months (HR=0.84, p=0.43) |
| Heudel et al, 2017 | Stage III-IV or recurrent endometrial cancer, received no more than 1 previous line of chemotherapy | PI3K inhibitor BKM120 | Not reported | 2.5 months |
| Pautier et al, 2016 | Advanced/metastatic or recurrent endometrial cancer | Irosustat | 8.3% | 16 weeks |
| McMeekin et al, 2015 | Locally advanced, recurrent, or metastatic endometrial cancer | Ixabepilone | 15.2% | Not reported |
| Emons et al, 2015 | Advanced and recurrent endometrial cancer | Temsirolimus | 10.0% | 3.0 months |
| Oza et al, 2015 | Metastatic or recurrent endometrial cancer with progressive disease after 1-2 lines of chemotherapy | Ridaforolimus | 0% (radiologic review)  8.3% (investigator evaluation) | 5.6 months |
| Konecny et al, 2015 | Advanced or metastatic endometrial cancer with progression after first line of chemotherapy | Dovitinib | Not reported | FGFR2^mut^ group: 4.1 months  FGFR2^non-mut^ group: 2.7 months |
| Lindemann et al, 2014 | Stage III-IV or recurrent endometrial cancer | Exemestane | ER+ group – 10% | Not reported |
| Emons et al, 2013 | Advanced or recurrent endometrial cancer | Fulvestrant | 11.4% | 2.3 months |
| Ray-Coquard et al, 2013 | Advanced or recurrent endometrial cancer refractory to 1-2 lines of chemotherapy | Everolimus | 5% of patients PR+ | 2.8 months |

**Table S2:** Current trials ongoing and published abstracts assessing response of RMEC to systemic therapies

| **Study** | **Study Population** | **Treatment Arms** | **Overall Response Rate** | **PFS** |
| --- | --- | --- | --- | --- |
| NCT03603184 | Advanced/recurrent endometrial cancer | Atezolizumab with paclitaxel and carboplatin  Placebo with paclitaxel and carboplatin | Trial ongoing | Trial ongoing |
| NCT03503786  MITO END-3 | Stage III-IV and recurrent endometrial cancer | Carboplatin and paclitaxel  Carboplatin and paclitaxel and avelumab | Trial ongoing | Trial ongoing |
| Mirza et al, 2021 (abstract) | Stage IIIC2, IVA and IVB or recurrent endometrial cancer | Nintedanib with carboplatin and paclitaxel | Not reported | 8.3 months |
| Mirza et al, 2020 (abstract)  NSGO-PALEO/ENGOT-EN3 trial | ER+ endometrial cancer evaluable/measurable by RECIST v1.1 | Palbociclib and letrozole  Letrozole and placebo | Not reported | Combination arm: 8.3 months  Letrozole alone: 3.0 months |
| Vergote et al, 2013 (abstract) | Recurrent endometrial cancer after 1 prior line of platinum-based chemotherapy | Lenvatinib | 14.3% by independent review, 21.8% by investigator assessment | 5.4 months |
| Pautier et al, 2012 (abstract) | Advanced/metastatic or recurrent ER+ endometrial cancer | Irosustat  Megestrol acetate | Irosustat: 11.1%  Megestrol acetate: 32.4% | Irosustat: 16 weeks (90% CI 9-34)  Megestrol acetate: 32 weeks (90% CI 16-63) |
| Miller et al, 2018 (Abstract) | Advanced/metastatic or recurrent endometrial cancer previously treated with carboplatin and paclitaxel | Zoptarelin | Not reported | 4.7 months |

**Table S3:** Data collection points

| Patient ID |
| --- |
| Date of birth |
| Date of initial diagnosis of endometrial cancer |
| Date of diagnosis of first recurrence or metastatic disease |
| Histology |
| Grade |
| Stage |
| Lymphvascular space invasion |
| Serosal invasion |
| Myometrial invasion |
| Size of uterine tumor |
| Cervical involvement |
| Adnexal involvement |
| Pelvic lymph node status |
| Para-aortic lymph node status |
| ER status (≥10% considered positive) |
| PR status (≥10% considered positive) |
| MSI status |
| Lynch testing |
| P53 status |
| Diabetes |
| BMI at diagnosis |
| Breast cancer history |
| Number of cycles of systemic treatment prior to progestin |
| Start date of progestin therapy |
| End date of progestin therapy |
| Type of progestin used |
| Dose of progestin used |
| Dose changes of progestin treatment |
| Date of diagnosis of progression of disease while on progestin therapy |
| Method of diagnosis of progression of disease |
| Date of death |
| Reason for stopping progestin |

1. **B)**


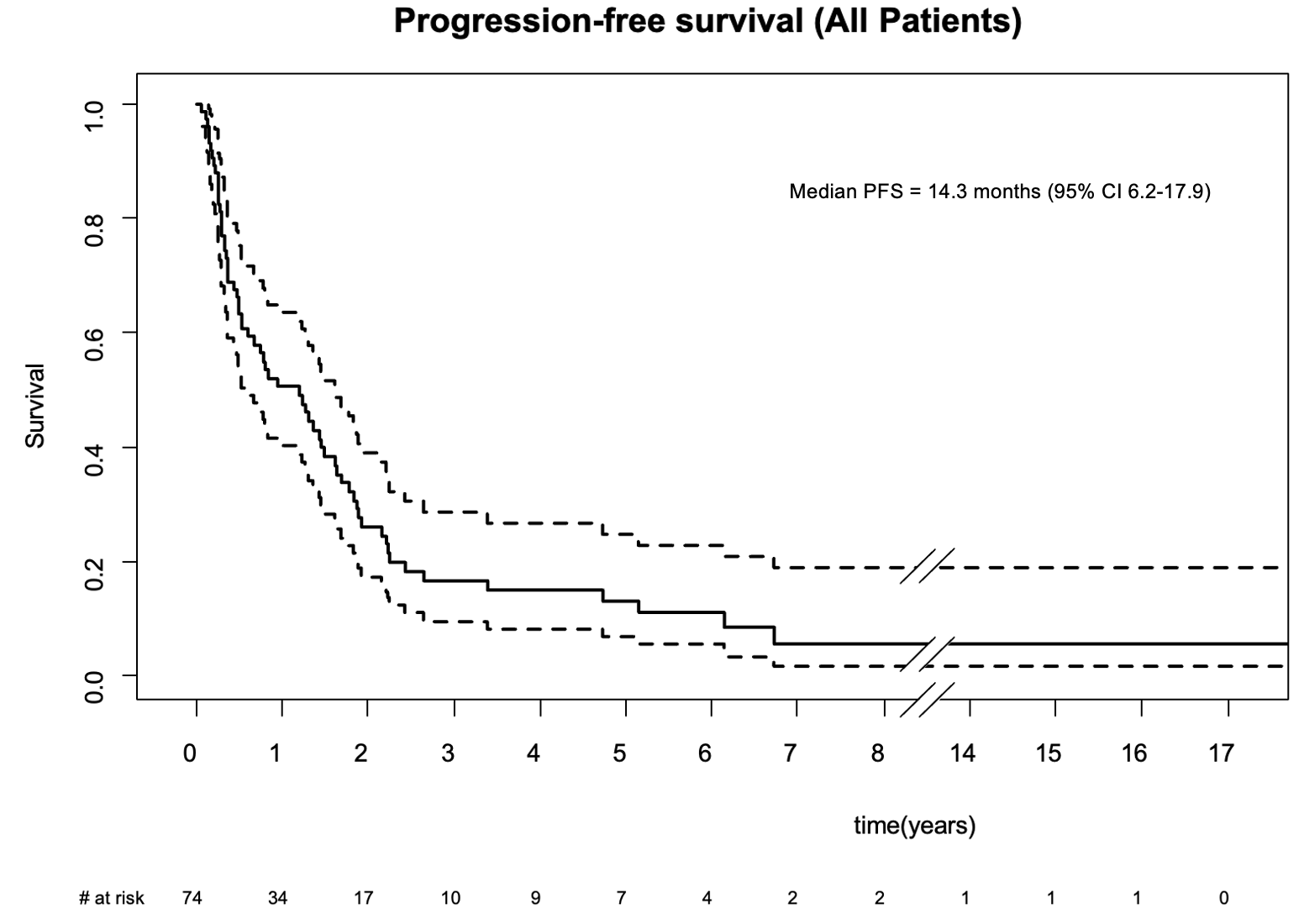

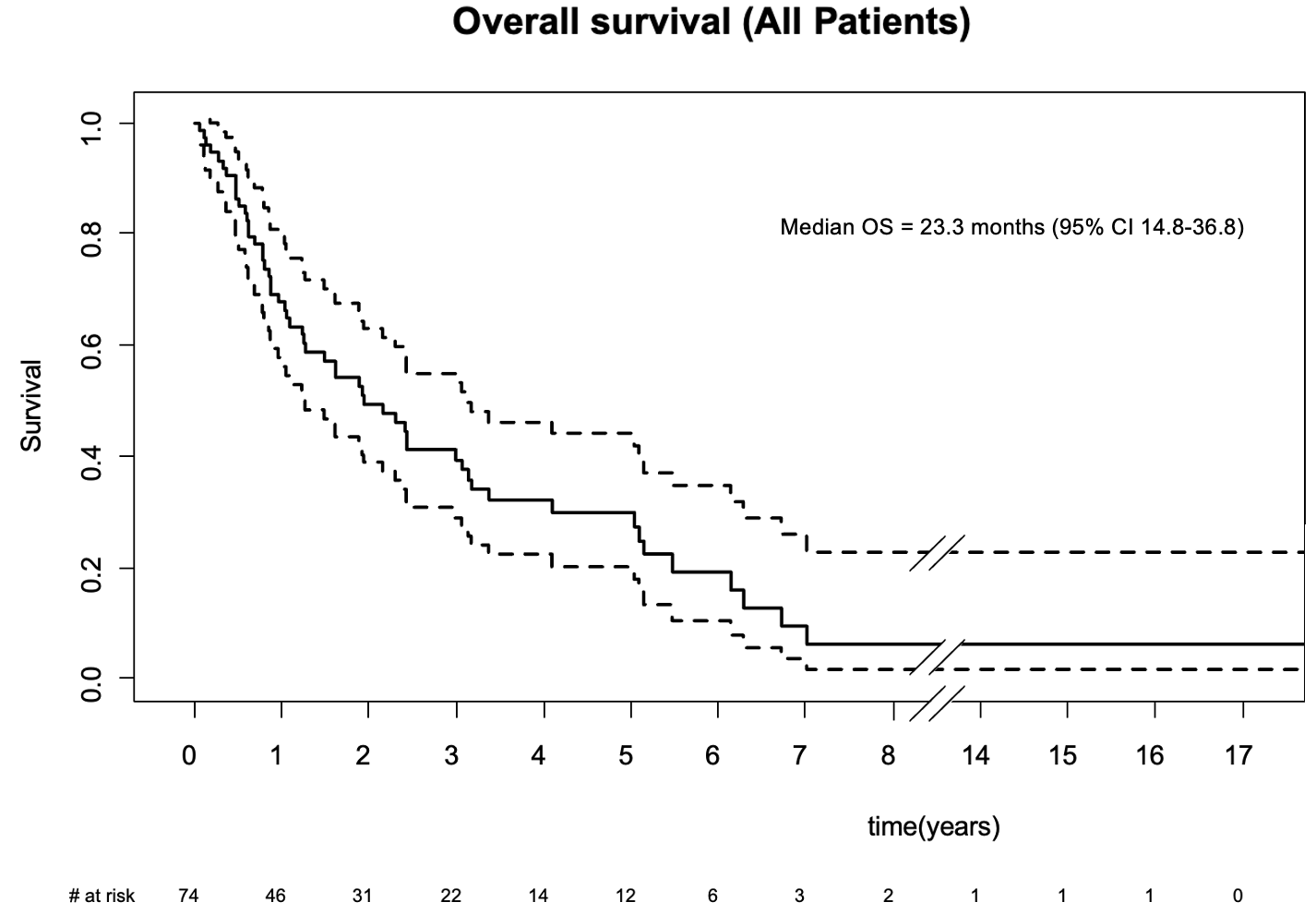


**Supplemental Figure 1:** Kaplan-Meier curves demonstrating A) PFS and B) OS for the total study sample.
